# Supplementary material for: Textural Features of Mouse Glioma Models Measured by Dynamic Contrast-Enhanced MR Images with 3D Isotropic Resolution
Source: Tomography. 2023 Mar 24;9(2):721–35. doi: 10.3390/tomography9020058 (PMC10141208; doi:10.3390/tomography9020058)
Supplement: Supplementary file 1 [file tomography-09-00058-s001.zip › tomography-2231693-supplementary.pdf]

tomography

MDPI

Supplementary Materials:

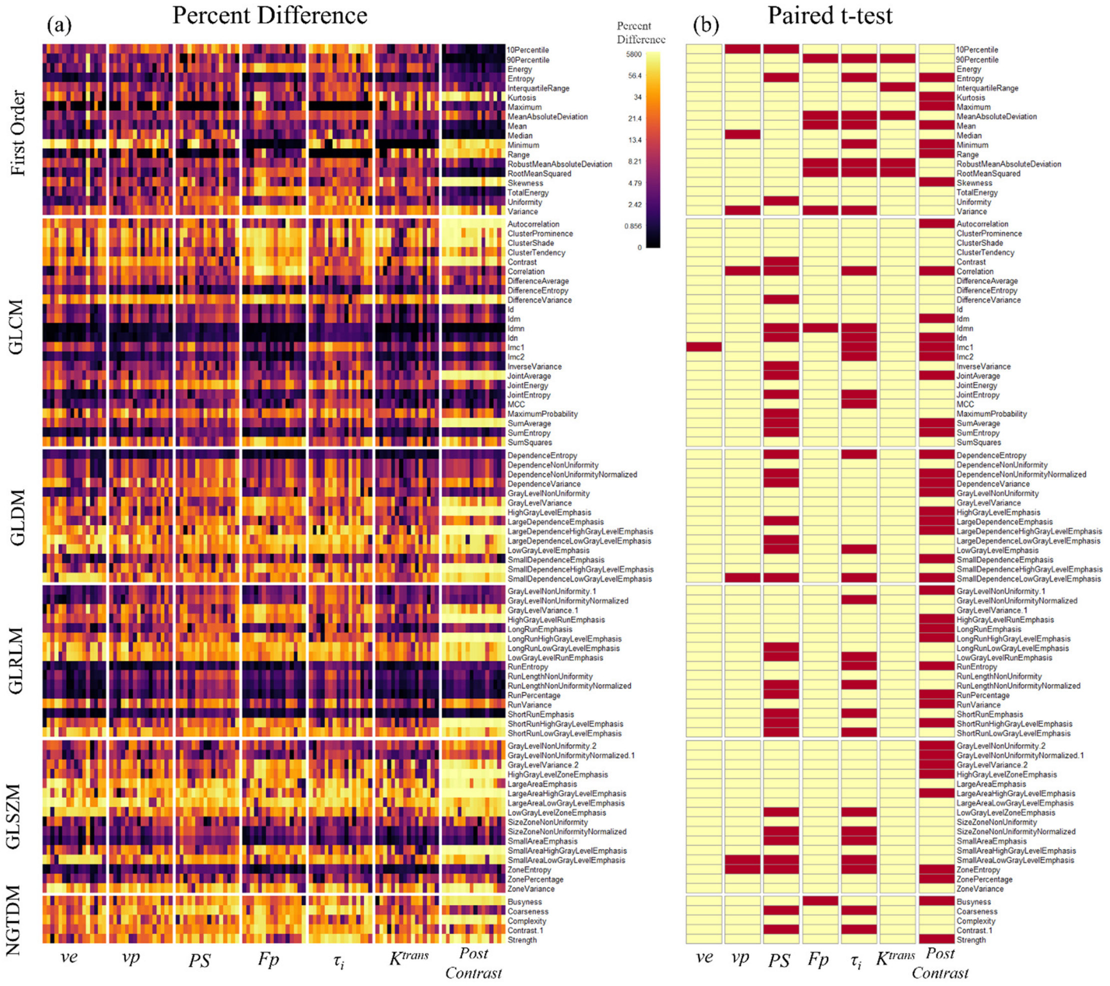

Figure S1. Percent difference and significance between features from isotropic and anisotropic (slice thickness 0.234 mm) images. (**a**) Heatmap depicting percent difference in histogram and texture features between isotropic parameter maps and up-sampled anisotropic maps (slice thickness 0.234 mm in axial direction). (**b**) Heatmap showing the features that have significant difference in radiomics features ($p < 0.00054$, depicted in red) between isotropic vs anisotropic image resolution, which are over 19.8% of all features.

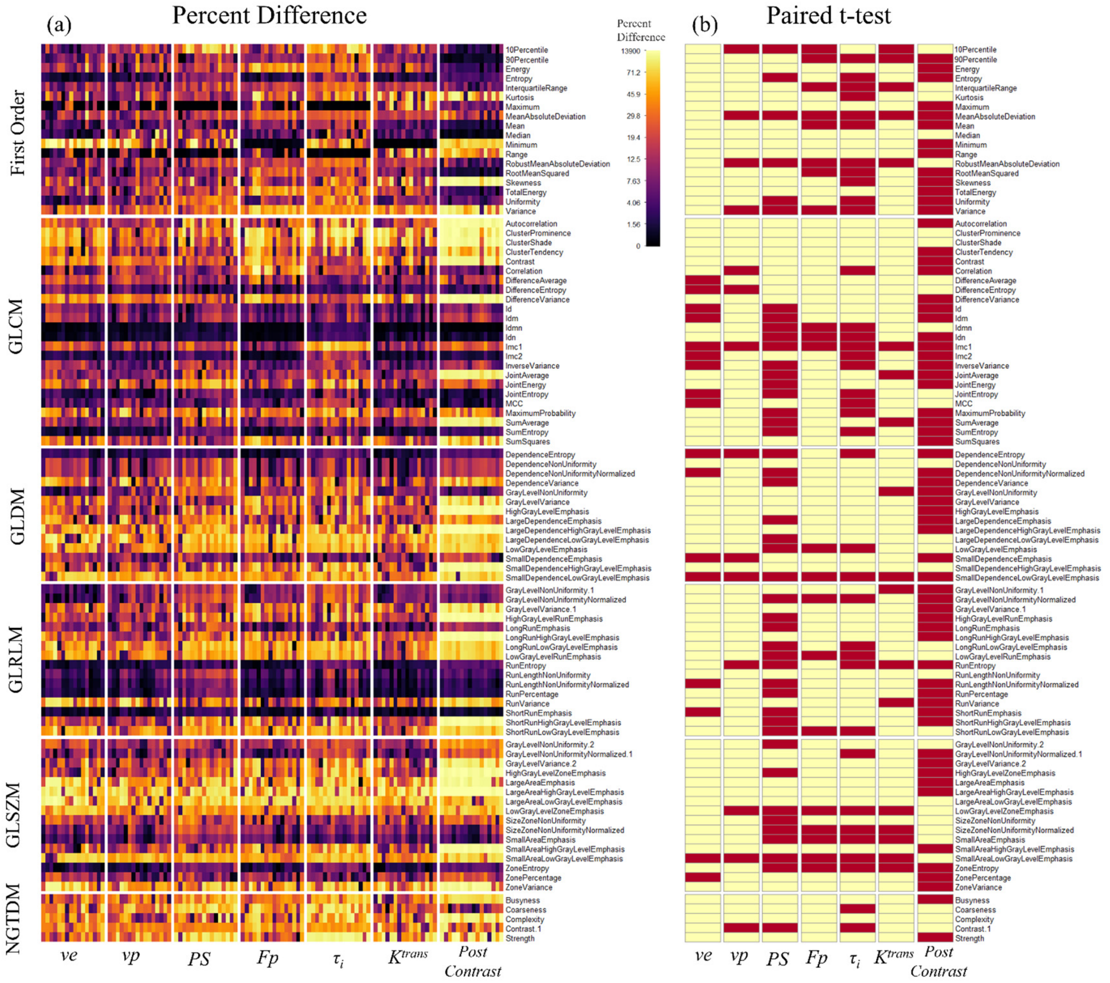

Figure S2. Percent difference and significance between features from isotropic and anisotropic (slice thickness 0.546 mm) images. (**a**) Heatmap depicting percent difference in histogram and texture features between isotropic parameter maps and up-sampled anisotropic maps (slice thickness 0.546 mm in axial direction). (**b**) Heatmap showing the features that have significant difference in radiomics features ($p < 0.00054$, depicted in red) between isotropic vs anisotropic image resolution, which are over 33.0% of all features.

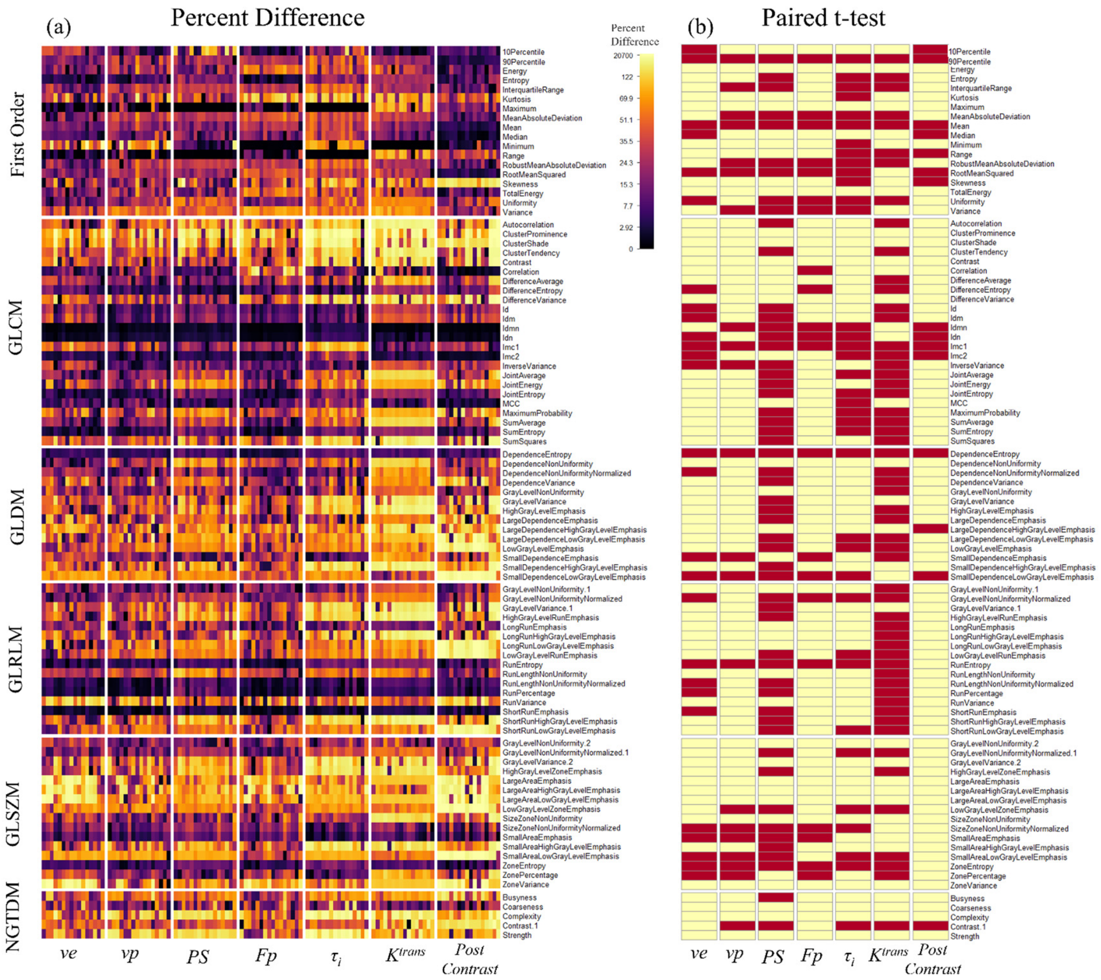

Figure S3. Percent difference and significance between features from isotropic and anisotropic (slice thickness 1.014 mm in coronal direction) images. (**a**) Heatmap depicting percent difference in histogram and texture features between isotropic parameter maps and up-sampled anisotropic maps (slice thickness 1.014 mm in coronal direction). (**b**) Heatmap showing the features that have significant difference in radiomics features ($p < 0.00054$, depicted in red) between isotropic vs anisotropic image resolution, which are over 35.3% of all features**.**

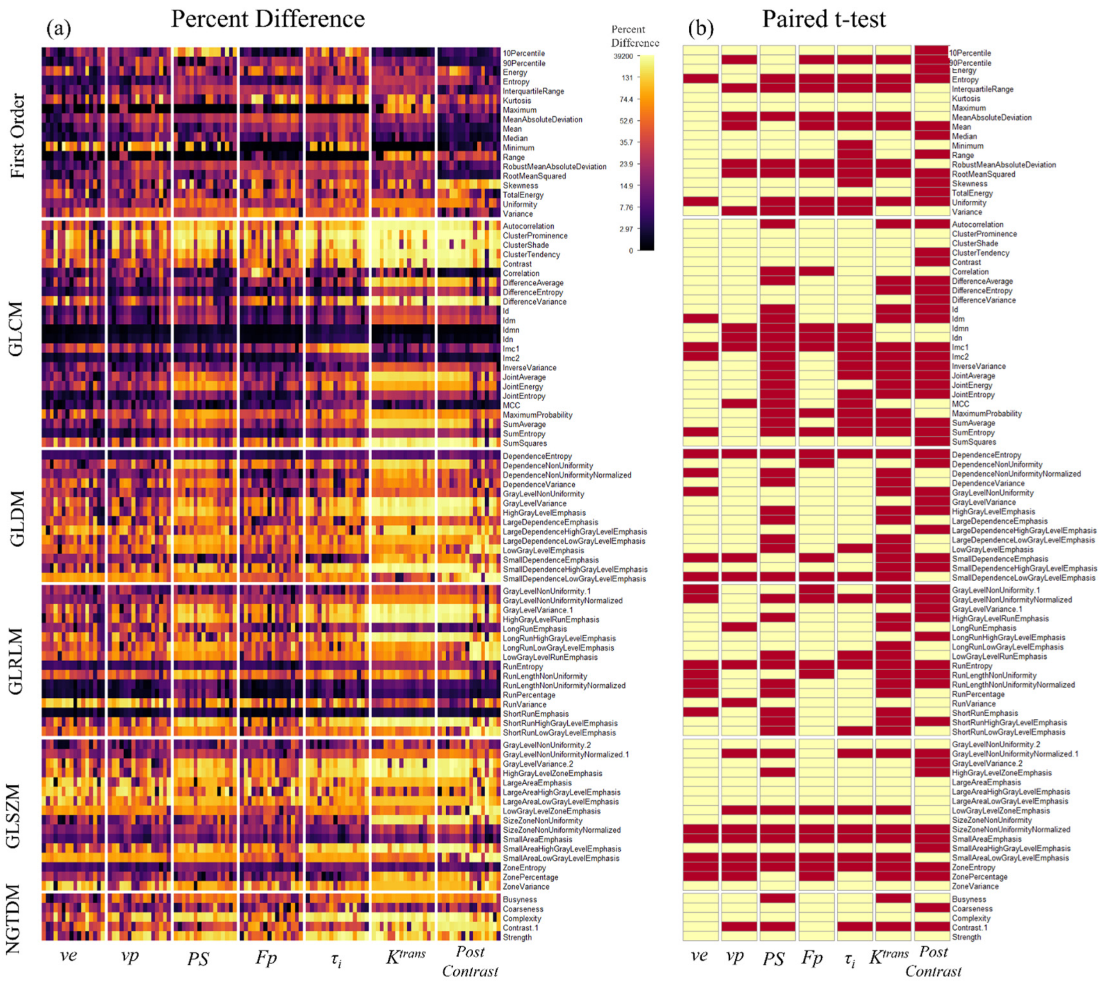

Figure S4. Percent difference and significance between features from isotropic and anisotropic (slice thickness 1.014 mm in sagittal direction) images. (**a**) Heatmap depicting percent difference in histogram and texture features between isotropic parameter maps and up-sampled anisotropic maps (slice thickness 1.014 mm in sagittal direction). (**b**) Heatmap showing the features that have significant difference in radiomics features ($p < 0.00054$, depicted in red) between isotropic vs anisotropic image resolution, which are over 42.1% of all features.
